# Potential Pharmacological Resources: Natural Bioactive Compounds from Marine-Derived Fungi

**DOI:** 10.3390/md14040076

**Published:** 2016-04-22

**Authors:** Liming Jin, Chunshan Quan, Xiyan Hou, Shengdi Fan

**Affiliations:** College of Life Science, Dalian Nationalities University, No. 18, LiaoHe West Road, Dalian 116600, China; jlm@dlnu.edu.cn (L.J.); xyhous@dlnu.edu.cn (X.H.); fsd@dlnu.edu.cn (S.F.)

**Keywords:** marine-derived fungi, fungal metabolites, bioactive compounds, natural products

## Abstract

In recent years, a considerable number of structurally unique metabolites with biological and pharmacological activities have been isolated from the marine-derived fungi, such as polyketides, alkaloids, peptides, lactones, terpenoids and steroids. Some of these compounds have anticancer, antibacterial, antifungal, antiviral, anti-inflammatory, antioxidant, antibiotic and cytotoxic properties. This review partially summarizes the new bioactive compounds from marine-derived fungi with classification according to the sources of fungi and their biological activities. Those fungi found from 2014 to the present are discussed.

## 1. Introduction

The oceans, which cover more than 70% of the earth’s surface and more than 95% of the earth’s biosphere, harbor various marine organisms. Because of the special physical and chemical conditions in the marine environment, almost every class of marine organism displays a variety of molecules with structurally unique features. However, unlike the long historical medical uses of terrestrial plants, marine organisms have a shorter history in pharmacological application [1]. In recent years, a significant number of novel metabolites with pharmacological potential have been discovered from marine organisms, such as polyketides, alkaloids, peptides, proteins, lipids, shikimates, glycosides, isoprenoids and hybrids, which exhibit biological activity including anticancer, antitumor, antiproliferative, antimicrotubule, cytotoxic, photo protective, as well as antibiotic and antifouling properties [2]. Among them, marine microorganisms, such as bacteria, actinomycetes, fungi and cyanobacteria have attracted more attention as potential lead compound producers. In comparison to marine invertebrates, they are a renewable and a reproducible source, as they can be cultured and can even be envisaged as amazing microbial factories for natural products [3].

Previously, scientists always focused on actinomycetes for their abilities to produce antibiotics. In fact, many fungal metabolites in the pharmaceutical market indicates the potential of microorganisms as valuable sources of lead drugs, e.g., the antibiotic polyketide griseofulvin (Likuden M^®^), the antibacterial terpenoid fusidic acid (Fucidine^®^), semi-synthetic or synthetic penicillins and cephalosporins, macrolides, statins as well as the ergot alkaloids such as ergotamine (Ergo-Kranit^®^) [4]. In 1949, the first secondary metabolite isolated from a marine-derived fungal strain, famous cephalosporin C, was produced by a culture of a *Cephalosporium* sp. isolated from the Sardinian coast. However, this was a more or less accidental discovery.

Despite the discovery of such important drug from marine fungi, the number of bioactive natural products originated from marine fungi increased extremely slowly. It is only from the late 1980s that researchers have focused on marine-derived fungi. In fact, marine-derived fungi are very important sources for novel bioactive secondary metabolites that could potentially be used as drugs. Blunt *et al.*, mentioned that marine-derived fungi have a greater proportion of marine natural compounds with more desirable oral-bioavailability and physico-chemical properties with molecular weight (MW) < 400 and clog*P* (calculated octanol-water log*P*) < 4 [5]. Compounds that meet these criteria can suggest the optimum combinations for potential pharmaceuticals [5]. Currently, thousands of structurally unique and biologically active compounds have been reported from marine fungi.

According to a classical definition, marine fungi are divided into obligate marine fungi and facultative marine fungi [6]. In fact, marine fungi often live as symbionts in algae, mangrove, coral, sea anemone, starfish, sea urchin, seagrass, and, especially, sponges. Collection of marine fungi usually requires the collection of the host or supporting material (e.g., algae, marine invertebrates, sediment or water, and even driftwood). Herein, a neutral term “marine-derived fungi” was used, which includes any fungal strain obtained from marine environment using cultivation techniques with “marine” media, which do not differentiate between facultative marine strains and contaminants from terrestrial habitats.

The aim of this review is to give an overview on secondary metabolites from marine-derived fungi and their biological activities, focusing on the period from 2014 to the present. In similar published reviews, assignments of a given metabolite to a certain category were generally based on structural considerations. It is obvious that classifications of the enormous structural diversity of marine fungal-derived metabolites are different in various literature reports, which only represents the authors’ personal judgments. In this article, these metabolites were classified according to the sources of marine fungi.

## 2. Metabolites from Marine-Derived Fungi

### 2.1. Marine Animals

#### 2.1.1. Sponge

**Figure 1:**
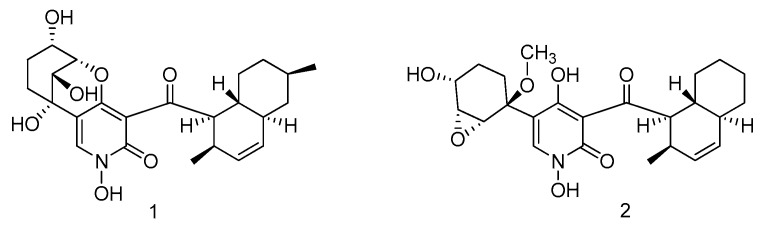
Structures of compounds **1**–**2**.

Two new 4-hydroxy-2-pyridone alkaloids, arthpyrones (**1**–**2**), were isolated from the fungus *Arthrinium arundinis* ZSDS1-F3, which obtained from sponge (Xisha Islands, China). Compounds **1** and **2** had significant *in vitro* cytotoxicities against the K562, A549, Huh-7, H1975, MCF-7, U937, BGC823, HL60, Hela and MOLT-4 cell lines, with IC_50_ values ranging from 0.24 to 45 μM. Furthermore, compound **2** displayed significant AchE inhibitory activity (IC_50_ = 0.81 μM), whereas compound **1** showed modest activity (IC_50_ = 47 μM) (Figure 1) [7].

**Figure 2:**
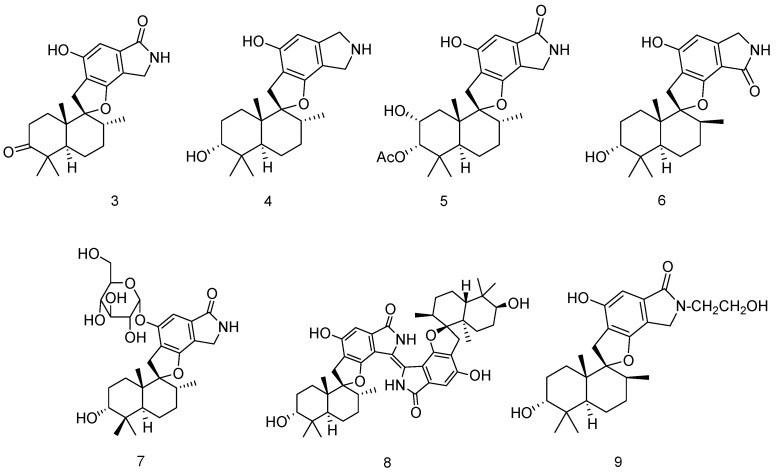
Structures of compounds **3**–**9**.

Chemical examination of the solid culture of the endophytic fungus *Stachybotrys chartarum* isolated from the sponge *Niphates recondita* (Weizhou Island in Beibuwan Bay, Guangxi Province of China) resulted in the isolation of seven new phenylspirodrimanes, named chartarlactams (**3**–**9**). Compounds **3**–**9** exhibited potent lipid-lowering effects in HepG2 cells in a dose of 10 μM (Figure 2) [8].

**Figure 3:**
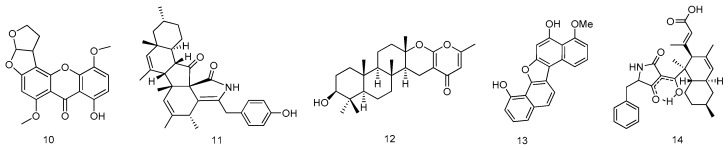
Structures of compounds **10**–**14**.

The extract of a strain of *Aspergillus versicolor* MF359 (from the sponge of *Hymeniacidon perleve*, Bohai Sea, China) yielded one new secondary metabolites, named 5-methoxydihydrosterigmatocystin (**10**). Compound **10** showed potent activity against *Staphylococcus aureus* (*S. aureus*) and *Bacillus subtilis* (*B. subtillis*) with MIC values of 12.5 and 3.125 μg/mL, respectively (Figure 3) [9].

The fungus *Diaporthaceae* sp. PSU-SP2/4 from marine sponge (Trang city, Thailand) generated a new pentacyclic cytochalasin (diaporthalasin, **11**). Compound **11** displayed potent antibacterial activity against both *S. aureus* and methicillin-resistant *S. aureus* (MRSA) with equal MIC values of 2 μg/mL (Figure 3) [10].

A new chevalone derivative, named chevalone E (**12**), was isolated from the ethyl acetate extract of the undescribed marine sponge-associated fungus *Aspergillus similanensis* KUFA 0013, which was collected from the Similan Islands, Phang Nga Province, Southern Thailand. Compound **12** was found to show synergism with the antibiotic oxacillin against methicillin-resistant *S. aureus* (Figure 3) [11].

Xylarianaphthol-1 (**13**), a new dinaphthofuran derivative, was isolated from an Indonesian marine sponge-derived fungus of order *Xylariales* on the guidance of a bioassay using the transfected human osteosarcoma MG63 cells (MG63^luc+^). Compound **13** activated p21 promoter stably transfected in MG63 cells with dose-dependent pattern. Expression of p21 protein in the wild-type MG63 cells was also promoted by xylarianaphthol-1 treatment, indicating compound **13** was expected to contribute to cancer prevention or treatment (Figure 3) [12].

A new polyketide with a new carbon skeleton, lindgomycin (**14**), was extracted from mycelia and culture broth of different Lindgomycetaceae strains, which were isolated from a sponge of the Kiel Fjord in the Baltic Sea (Germany) and from the Antarctic. Compound **14** showed antibiotic activities with IC_50_ value of 5.1 (±0.2) μM against MRSA (Figure 3) [13].

#### 2.1.2. Coral

**Figure 4:**
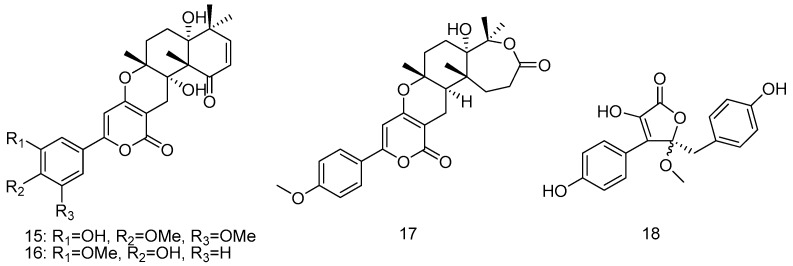
Structures of compounds **15**–**18**.

The fungus *Aspergillus terreus* SCSGAF0162 was isolated from gorgonian corals *Echinogorgia aurantiaca* (the South China Sea). Three lactones including three territrem derivatives (**15**–**17**) and a butyrolactone derivative (**18**) were isolated from the fungus under solid-state fermentation of rice. Among them, compounds **15** and **16** showed strong inhibitory activity against acetylcholinesterase with IC_50_ values of 4.2 ± 0.6 and 4.5 ± 0.6 μM, respectively. This was the first report that compounds **17** and **18** had evident antiviral activity towards HSV-1, with IC_50_ values of 16.4 ± 0.6 and 21.8 ± 0.8 μg·mL^–1^, respectively. Moreover, compound **15** had obvious antifouling activity with EC_50_ values of 12.9 ± 0.5 μg·mL^–1^ toward barnacle *Balanus amphitrite* larvae (Figure 4) [14].

**Figure 5:**
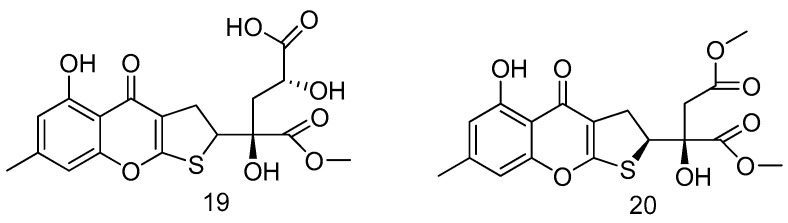
Structures of compounds **19**–**20**.

Two new dihydrothiophene-condensed chromones, oxalicumones (**19**–**20**) were isolated from a culture broth of the marine gorgonian-associated fungus *Penicillium oxalicum* SCSGAF 0023. Compounds **19** and **20** showed significant cytotoxicity against several carcinoma cell lines with IC_50_ less than 10 μM (Figure 5) [15].

**Figure 6:**
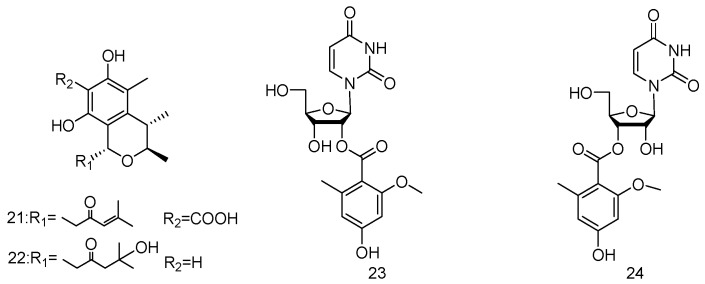
Structures of compounds **21**–**24**.

The fungal strain *Nigrospora oryzae* SCSGAF 0111 (from marine gorgonian *Verrucella umbraculum*, South China Sea) yielded two new citrinins, nigrospins B and C (**21**–**22**). Compounds **21**–**22** showed weak antifungal activity against *Aspergillus versicolor* with inhibition zone of 8 cm at 50 μg/paper disc, with a positive control thiram of 8 cm at 5 μg/paper disc (Figure 6) [16].

Two nucleoside derivatives (**23**–**24**) were isolated from the fungus *Aspergillus* *versicolor* which was derived from the gorgonian *Dichotella gemmacea* in the South China Sea. Compounds **23**/**24** (a mixture of compound **23**:compound **24** at a ratio of 7:10) exhibited selective antibacterial activity against *Staphylococcus epidermidis* with an MIC value of 12.5 μM (Figure 6) [17].

**Figure 7:**
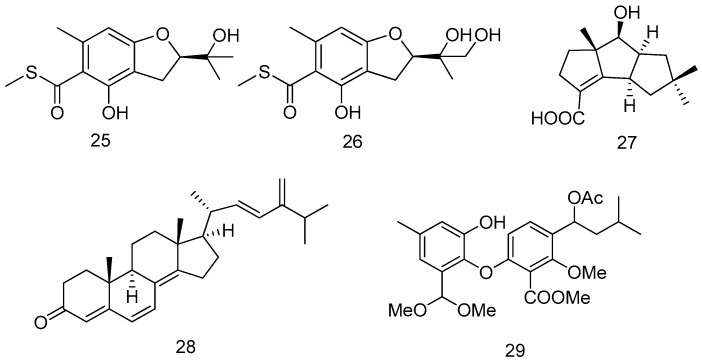
Structures of compounds **25**–**29**.

Two new sulfur-containing benzofuran derivatives, eurothiocin A and B (**25** and **26**) were isolated from the fungus *Eurotium rubrum* SH-823 which was obtained from a *Sarcophyton* sp. soft coral in the South China Sea. The compounds (**25** and **26**) shared a methyl thiolester moiety, which was quite rare in natural secondary metabolites. Both of them exhibited more potent inhibitory effects against α-glucosidase activity than acarbose, which was the clinical α-glucosidase inhibitor. Further mechanistic analysis demonstrated that both of them exhibited competitive inhibition characteristics (Figure 7) [18].

*Chondrostereum* sp. was isolated from the inner tissue of a soft coral *Sarcophyton tortuosum*, which was collected from the Hainan Sanya National Coral Reef Reserve, China. When this fungus was cultured in a liquid medium containing glycerol as the carbon source, a new metabolite, chondrosterin **27** was obtained. Compound **27** exhibited potent cytotoxic activities against the cancer cell lines CNE-1 and CNE-2 with the IC_50_ values of 1.32 and 0.56 μM (Figure 7) [19].

A steroid derivative, compound **28** was isolated from the fermentation broth of a gorgonian-derived *Aspergillus* sp. fungus. The fungus was isolated from the inner part of the fresh gorgonian *M. abnormalis*, which was collected from the Xisha Islands coral reef of the South China Sea. Compound **28** inhibited the larval settlement of barnacle *Balanus amphitrite* with EC_50_ 18.40 ± 2.0 μg/mL (Figure 7) [20].

A new diphenyl ether derivative, talaromycin A (**29**) was isolated from a gorgonian-derived fungus, *Talaromyces* sp. The fungal strain was isolated from a piece of fresh tissue from the inner part of the gorgonian *Subergorgia suberosa*, collected from the Weizhou coral reef in the South China Sea. Compound **29** showed potent antifouling activities against the larval settlement of the barnacle *Balanus amphitrite* with the EC_50_ value 2.8 ± 0.2 μg/mL (Figure 7) [21].

#### 2.1.3. Starfish

**Figure 8:**
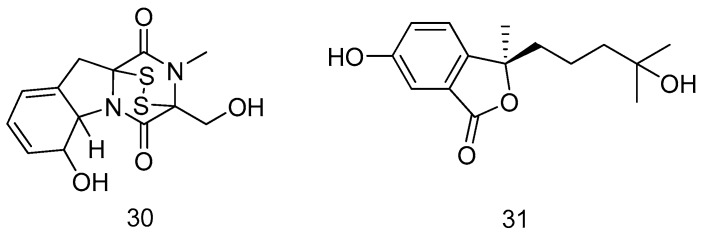
Structures of compounds **30**–**31**.

Liang *et al.* [22] investigated the influence on secondary metabolites with variety of cultivation parameters of marine fungus, *Neosartorya pseudofischeri*, which was isolated from the inner tissue of starfish *Acanthaster planci*. Glycerol-peptone-yeast extract (GlyPY) and glucose-peptone-yeast extract (GluPY) media were applied to culture this fungus. A novel gliotoxin (**30**) was produced with GluPY medium. Compound **30** displayed significant inhibitory activities against three multidrug-resistant bacteria, *S. aureus* (ATCC29213), MRSA (R3708) and *Escherichia coli* (*E. coli*) (ATCC25922), as well as cytotoxicities against some cell lines including human embryonic kidney (HEK) 293 cell line and human colon cancer cell lines, HCT-116 and RKO (a poorly differentiated colon carcinoma cell line) (Figure 8).

A novel isobenzofuranone derivative, pseudaboydins A (**31**) was isolated from the marine fungus, *Pseudallescheria boydii*, associated with the starfish, *Acanthaster planci*. Compound **31** showed moderate cytotoxic activity against HONE1, SUNE1 and GLC82 with IC_50_ values of 37.1, 46.5 and 87.2 μM, respectively (Figure 8) [23].

#### 2.1.4. Bryozoan

**Figure 9:**
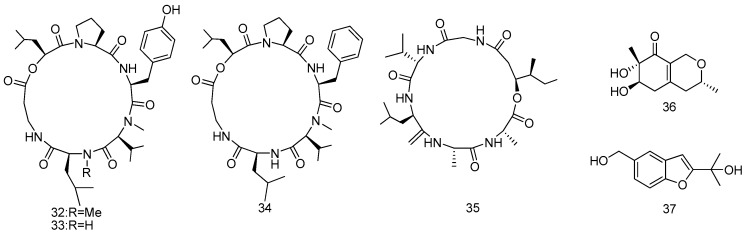
Structures of compounds **32**–**37**.

Three new cyclohexadepsipeptides of the isaridin class including isaridin G (**32**), desmethylisaridin G (**33**), and desmethylisaridin C1 (**34**) were isolated and identified from the marine bryozoan-derived fungus *Beauveria felina* EN-135. Compounds **32**–**34** showed inhibitory activity against *E.coli* with MIC values of 64, 64, and 8 μg/mL, repectively. This is the first report on antibacterial activities of the isaridins (Figure 9) [24].

Bioassay-guided fractionation of a culture extract of *Beauveria felina* EN-135, an entomopathogenic fungus isolated from an unidentified marine bryozoan, led to the isolation of a new cyclodepsipeptide, iso-isariin D (**35**); two new *O*-containing heterocyclic compounds felinones A and B (**36** and **37**). Compound **35** exhibited potent lethality against brine shrimp (*Artemia salina*), with LD_50_ values of 26.58 μΜ, notably stronger than that of the positive control colchicine, while compounds **36** and **37** possessed weak activity. Only compound **37** showed inhibitory activity (MIC value of 32 μg/mL) higher than that of the chloramphenicol control (MIC value of 4 μg/mL) against *Pseudomonas aeruginosa* (Figure 9) [25].

#### 2.1.5. Sea Urchin

**Figure 10:**
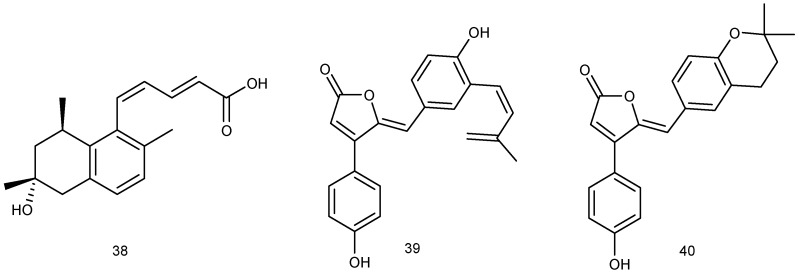
Structures of compounds **38**–**40**.

The *Penicillium* sp. SF-6013 was isolated from the sea urchin *Brisaster latifrons*, which was collected from the Sea of Okhotsk. Chemical investigation of strain SF-6013 resulted in the discovery of a new tanzawaic acid derivative, 2*E*,4*Z*-tanzawaic acid D (**38**). Screening for anti-inflammatory effects in lipopolysaccharide (LPS)-activated microglial BV-2 cells indicated that compound **38** inhibited the production of nitric oxide (NO) with IC_50_ values of 37.8 μM (Figure 10) [26].

#### 2.1.6. Fish

Two new rubrolides, rubrolides R (**39**) and S (**40**), were isolated from the fermentation broth of the marine-derived fungus *Aspergillus terreus* OUCMDZ-1925, which was isolated from the viscera of *C. haematocheilus* grown in the waters of the Yellow River Delta. Compound **39** showed comparable or superior antioxidation against ABTS radicals to those of trolox and ascorbic acid with an IC_50_ value of 1.33 μM. Compound **40** showed comparable or superior anti-influenza A (H1N1) virus activity to that of ribavirin with an IC_50_ value of 87.1 μM. Compounds **39** and **40** showed weak cytotoxicity against the K562 cell line with IC_50_ values of 12.8 and 10.9 μM, respectively, while were inactive against the A549, HL-60, Hela and HCT-116 cell lines (Figure 10) [27].

#### 2.1.7. Prawn

**Figure 11:**
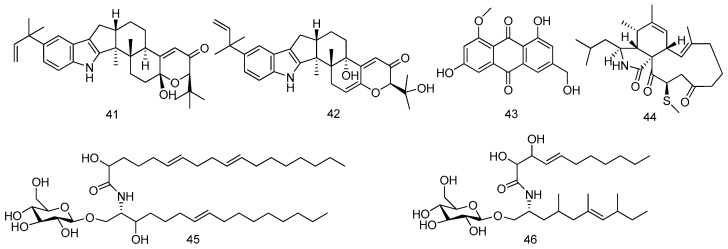
Structures of compounds **41**–**46**.

The fungal strain, *Aspergillus flavus* OUCMDZ-2205, was obtained from the prawn, *Penaeus vannamei*, from the Lianyungang sea area, Jiangsu Province of China. Two new indole-diterpenoids (**41** and **42**) were isolated from the fermentation broth of the fungus. Compound **41** exhibited antibacterial activity against *S. aureus* with a MIC value of 20.5 μM and showed PKC-beta inhibition with an IC_50_ value of 15.6 μM. Both **41** and **42** could arrest the A549 cell cycle in the S phase at a concentration of 10 μM (Figure 11) [28].

#### 2.1.8. Others

The marine-derived fungus *Eurotium amstelodami* was isolated from an unidentified marine animal collected from the Sungsan coast in Jeju Island, Korea. An anthraquinone derivative, questinol (**43**) was successfully isolated from the broth extract of the fungus for the first time. Questinol (**43**) did not exhibit cytotoxicity in LPS-stimulated RAW 264.7 cells up to 200 μM while could significantly inhibit NO and PGE_2_ production at indicated concentrations. Furthermore, it could inhibit the production of pro-inflammatory cytokines, including IL-1β, TNF-α, and IL-6 and suppress the expression level of iNOS in a dose-dependent manner through the western blot analysis. All these results suggest that questinol might be selected as a promising agent for the prevention and therapy of inflammatory disease (Figure 11) [29].

A novel aspochalasin, 20-β-methylthio-aspochalsin Q (named as aspochalasin V, **44**) was isolated from culture broth of *Aspergillus* sp., which was obtained in the gut of a marine isopod *Ligia oceanica* (Dinghai in Zhoushan, Zhejiang Province of China). This is the first report about methylthio-substituted aspochalasin derivative. Apochalasin V showed moderate cytotoxic activity against the prostate cancer PC3 cell line and HCT116 cell line with IC_50_ values of 30.4 and 39.2 μM, respectively (Figure 11) [30].

Two new cerebrosides, penicillosides A (**45**) and B (**46**) were isolated from the marine-derived fungus *Penicillium* species, which were gained from the Red Sea tunicate, *Didemnum* species in the Mangrove. Penicilloside A displayed antifungal activity against *Candida albicans* while penicilloside B illustrated antibacterial activities against *S. aureus* and *E. coli*. Additionally, both compounds showed weak activity against HeLa cells (Figure 11) [31].

### 2.2. Mangrove

**Figure 12:**
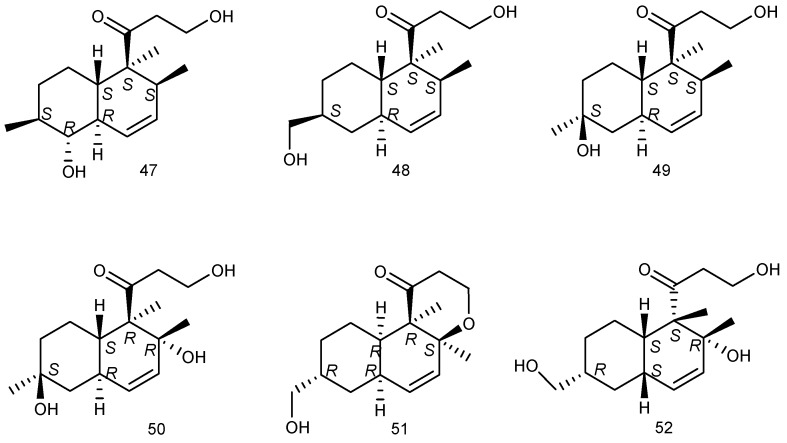
Structures of compounds **47**–**52**.

Six new compounds with polyketide decalin ring, peaurantiogriseols A–F (**47**–**52**), were isolated from the fermentation products of mangrove endophytic fungus *Penicillium aurantiogriseum*328#, which was collected from the bark of *Hibiscus tiliaceus* in the Qi’ao Mangrove Nature Reserve of Guangdong Province, China. Compounds **47**–**52**, showed low inhibitory activity against human aldose reductase (at a concentration of 50 µM), the corresponding value of percent inhibitions were 16%, 6%, 31%, 22%, 26%, 2%, respectively (Figure 12) [32].

**Figure 13:**
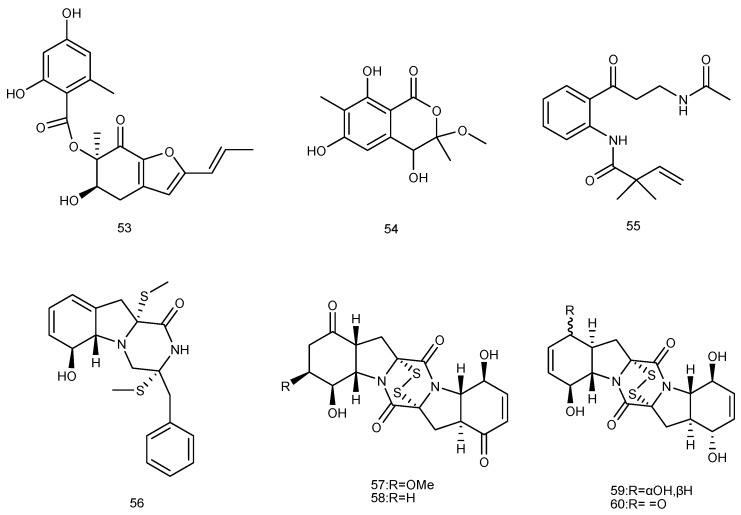
Structures of compounds **53**–**60**.

Aspergifuranone (**53**), isocoumarin derivatives (±) **54** were separated from the mangrove endophytic fungus *Aspergillus* sp. 16-5B, which was isolated from the leaves of *Sonneratia apetala* from Dongzhaigang Mangrove National Nature Reserve in Hainan Island, China. Both of them were evaluated for their α-glucosidase inhibitory activities, and compound **53** showed significant inhibitory activity with IC_50_ value of 9.05 ± 0.60 μM. Kinetic analysis showed that compound **53** was a noncompetitive inhibitor of α-glucosidase. Compound **54** exhibited moderate inhibitory activities, with IC_50_ value of 90.4 ± 2.9 μM (Figure 13) [33].

A new alkaloid, brocaeloid B (**55**), containing C-2 reversed prenylation, was isolated from cultures of *Penicillium brocae* MA-192, an endophytic fungus obtained from the fresh leaves of the marine mangrove plant *Avicennia marina* in Hainan island, China. Compound **55** showed lethality against brine shrimp (*Artemia salina*) with an LD_50_ value of 36.7 μM (Figure 13) [34].

The fungus *Phoma* sp. OUCMDZ-1847, which was isolated from a mangrove fruit sample of *Kandelia candel* (Wenchang, Hainan Province, China), generated a new thiodiketopiperazine, named phomazines B (**56**). Compound **56** showed cytotoxicity against the MGC-803 cell line with IC_50_ value of 8.5 μM (Figure 13) [35].

Four new disulfide-bridged diketopiperazine derivatives, brocazines (**57**–**60**) were isolated from *Penicillium brocae* MA-231, a fungus obtained from the fresh tissue of the marine mangrove plant *Avicennia marina* that was collected at Hainan Island, China. Compounds **57**–**60** showed cytotoxic activities against nine tumor cell lines, including Du145, HepG2, HeLa, NCI-H460, MCF-7, SGC-7901, SW1990, U251 and SW480, with IC_50_ values ranging from 0.89 to 9.0 μM (Figure 13) [36].

**Figure 14:**
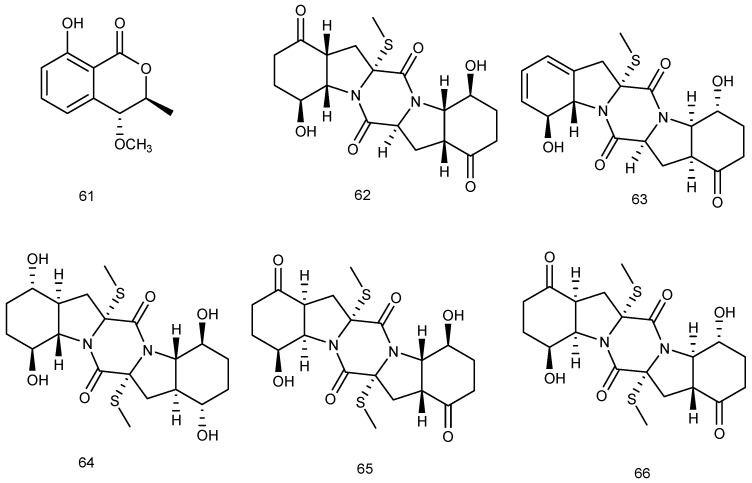
Structures of compounds **61**–**66**.

A new isochroman, (3*R*,4*S*)-3,4-dihydro-8-hydroxy-4-methoxy-3-methylisocoumarin (**61**), was isolated from the marine fungus *Phomopsis* sp. (No. Gx-4), which was obtained from the mangrove sediment of ZhuHai, Guangdong, China. Primary bioassays and preliminary pharmacological tests indicated that compound **61** could accelerate the growth of subintestinal vessel plexus (SIV) branches markedly (Figure 14) [37].

*Penicillium brocae* MA-231, an endophytic fungus, was obtained from the fresh tissue of the marine mangrove plant *Avicennia marina.* After investigation, five new sulfide diketopiperazine derivatives, namely, penicibrocazines A–E (**62**–**66**) were isolated and identified*.* In the antimicrobial experiments, compounds **63**–**65** showed activity against *S. aureus*, with MIC values of 32.0, 0.25 and 8.0 μg/mL, respectively. Compound **64** also showed activity against *Micrococcus luteus* with MIC value of 0.25 μg/mL. Moreover, compounds **63**, **65** and **66** implied activity against plant pathogen *Gaeumannomyces graminis* with MIC values of 0.25, 8.0, and 0.25 μg/mL, respectively (Figure 14) [38].

**Figure 15:**
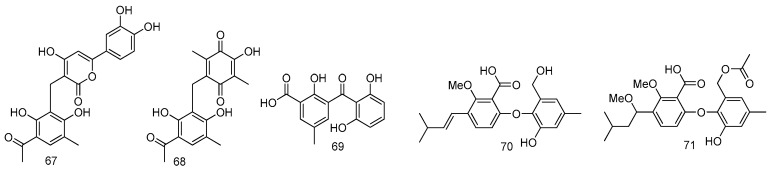
Structures of compounds **67**–**71**.

Two new biogenetically related compounds (**67**–**68**) have been isolated from a fungus *Penicillium* *dipodomyicola* HN4-3A from mangrove of South China Sea. Compounds **67** and **68** showed strong inhibitory activity against *Mycobacterium tuberculosis* protein tyrosine phosphatase B (MptpB) with IC_50_ values of 0.16 ± 0.02 μM and 1.37 ± 0.05 μM, respectively (Figure 15) [39].

Investigation of the marine mangrove-derived fungal strain *Penicillium* sp. MA-37 resulted in the isolation of one new benzophenone, *iso*-monodictyphenone (**69**) and two new diphenyl ether derivatives penikellides A (**70**) and B (**71**). Compounds **69**–**71** exhibited brine shrimp lethality, with LD_50_ values of 25.3, 14.2 and 39.2 μM, respectively, while the positive control colchicine had LD_50_ value of 1.22 μM. Compound **69** showed antibacterial activity against *Aeromonas hydrophilia* with MIC 8 μg/mL, while the positive control, chloromycetin exhibited a MIC of 4 μg/mL (Figure 15) [40].

**Figure 16:**
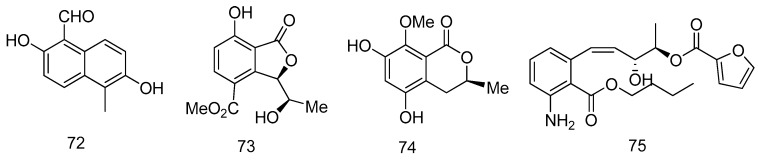
Structures of compounds **72**–**75**.

A new naphthalene derivative, vaccinal A (**72**), was isolated from *Pestalotiopsis vaccinii* (cgmcc3.9199) endogenous with the mangrove plant *Kandelia candel* (L.) Druce (Rhizophoraceae). Compound **72** exhibited *in vitro* anti-enterovirus 71 (EV71) with IC_50_ value of 19.2 μM and potent COX-2 inhibitory activity with IC_50_ value of 1.8 μM (Figure 16) [41].

The fungus *Astrocystis* sp. BCC 22166 was isolated from a mangrove palm, Nypa, at Hat Khanom-Mu Ko Thale Tai National Park, Nakhon Si Thammarat Province of Thailand. Two new compounds, phthalide **73**, dihydroisocoumarin **74** were separated from the fungus. Compound **73** exhibited antibacterial activity against *Bacillus cereus* (IC_50_ = 12.5 μg/mL), while compound **74** showed cytotoxicity to KB and Vero cells with values of IC_50_ 22.6 and 48.2 μg/mL respectively (Figure 16) [42].

A new aromatic amine, pestalamine A (**75**) was isolated from mangrove-derived endophytic fungus *Pestalotiopsis vaccinii* that was isolated from a branch of *Kandelia candel* (L.) Druce (*Rhizophoraceae*), a usual viviparous mangrove species in coastal and estuarine areas of southern China. Pestalamine A (**75**) showed moderate cytotoxicities against human cancer cell lines (MCF-7, HeLa, and HepG2) with IC_50_ values of 40.3, 22.0, and 32.8 μM, respectively (Figure 16) [43].

**Figure 17:**
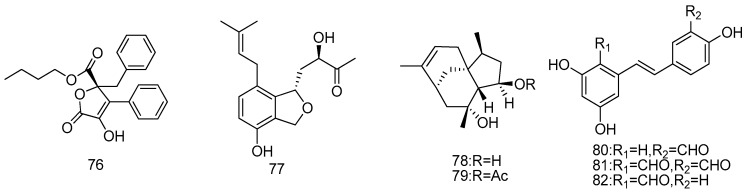
Structures of compounds **76**–**82**.

A new aromatic butyrolactone, flavipesins A (**76**), was isolated from marine-derived endophytic fungus *Aspergillus flavipes*. AIL8. This was isolated from the inner leaves of mangrove plant *Acanthus ilicifolius* (Daya Bay, Shenzhen City, Guangdong Province, China). Compound **76** displayed significant antibacterial activity against *S. aureus* (MIC = 8.0 μg/mL) and *B. subtillis* (MIC = 0.25 μg/mL). Compound **76** also showed the unique antibiofilm activity of decreasing the number of living cells embed in the biofilm matrix from 390.6 to 97.7 μg/mL (*p* < 0.01). This indicates that compound **76** could penetrate the biofilm matrix and kill the living bacteria inside mature *S. aureus* biofilm (Figure 17) [44].

A new prenylated phenol vaccinol I (**77**) was isolated from endogenous fungi *Pestalotiopsis vaccinii* (cgmcc3.9199) of mangrove plant *Kandelia candel* (L.) Druce (*Rhizophoraceae*). Compound **77** exhibited potent COX-2 inhibitory activity (IC_50_ = 16.8 μM) (Figure 17) [45].

Penicibilaenes A (**78**) and B (**79**), two sesquiterpenes possessing a tricyclo[6.3.1.0^1,5^] dodecane skeleton, were characterized from *Penicillium bilaiae* MA-267, a fungus obtained from the rhizospheric soil of the mangrove plant *Lumnitzera racemosa*. Both of them exhibited selective activity against the plant pathogenic fungus *Colletotrichum gloeosporioides* (MIC = 1.0 and 0.125 μg/mL, respectively) (Figure 17) [46].

Three new resveratrol derivatives, resveratrodehydes A–C (**80**–**82**), were isolated from the mangrove endophytic fungus *Alternaria* sp. R6. All compounds showed broad-spectrum inhibitory activities against human breast MDA-MB-435, human liver HepG2, and human colon HCT-116 by MTT assay (IC_50_ < 50 μM). Especially, compounds **80** and **81** both exhibited marked cytotoxic activities against HCT-116 and MDA-MB-435 cell lines (IC_50_ < 10 μM). Additionally, compounds **80** and **82** showed moderate antioxidant effect by DPPH radical scavenging assay (Figure 17) [47].

**Figure 18:**
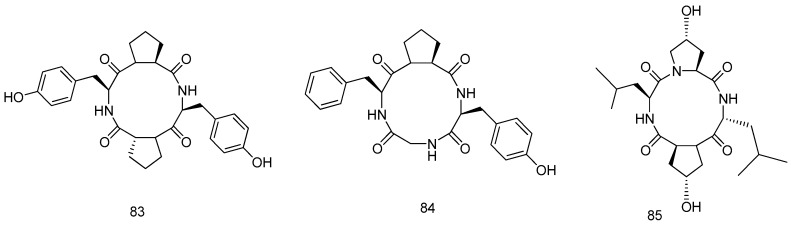
Structures of compounds **83–85**.

The strategy that co-cultivation of two mangrove fungi, *Phomopsis* sp*.* K38 and *Alternaria* sp. E33 (Leizhou Peninsula, Guangdong Province, China) in a single confined environment generated new active natural products, including three new cyclic tetrapeptides, cyclo(d-Pro-l-Tyr-l-Pro-l-Tyr) (**83**), cyclo(Gly-l-Phe-l-Pro-l-Tyr) (**84**) and cyclo(l-leucyl-*trans*-4-hydroxy-l-prolyl-d-leucyl-*trans*-4-hydroxy-l-proline) (**85**). Compounds **83**–**85** showed moderate to high antifungal activities (*Candida* *albicans*, *Gaeumannomyces* *graminis*, *Rhzioctonia* *cerealis*, *Helminthosporium* *sativum* and *Fusarium* *graminearum*) as compared with the positive control (Figure 18) [48,49].

### 2.3. Sediment

**Figure 19:**
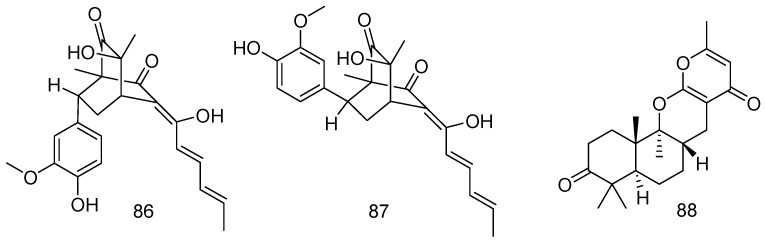
Structures of compounds **86**–**88**.

*Penicillium chrysogenum* PJX-17, which was separated from marine sediment (South China Sea) generated two novel sorbicillinoids combining a bicyclo[2.2.2] octane with a 2-methoxyphenol moiety, sorbicatechols A (**86**) and B (**87**) respectively. Compounds **86** and **87** exhibited activities against influenza virus A (H1N1), with IC_50_ values of 85 and 113 μM, respectively (Figure 19) [50].

The fungal strain *Penicillium* sp. F446, which was isolated from marine sediments at the depth of 25 m collected from Geomun-do (Island), Korea, generated a novel meroterpenoid, penicillipyrones B (**88**). Compound **88** showed significant induction of quinone reductase (Figure 19) [51].

**Figure 20:**
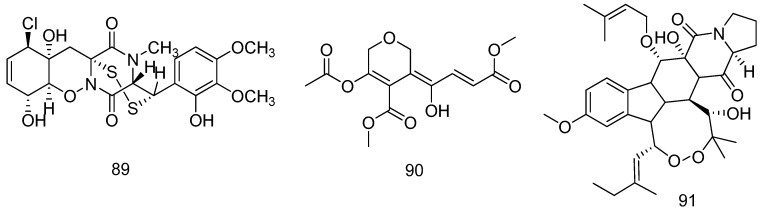
Structures of compounds **89**–**91**.

An epidithiodiketopiperazine, *N-*methyl-pretrichodermamide B (**89**) was isolated from the fungus *Penicillium* sp. WN-11-1-3-1-2, derived from the sediment of a hyper saline lake located at Wadi El-Natrun in Egypt, 80 km northwest of Cairo. Compound **89** showed pronounced cytotoxicity against the murine lymphoma L5178Y mouse lymphoma cell line, IC_50_ = 2 μM (Figure 20) [52].

One new polyketide (**90**) was isolated from the lipophilic extract of the marine-derived fungus *Isaria felina* KMM 4639 from marine sediments at a depth of 10 m (South China Sea, coast of Vietnam). Compound **90** exhibited cytotoxicity against HL-60 and THP-1 cell lines with IC_50_ values of 4.3 and 37.4 μM, respectively (Figure 20) [53].

One new indolediketopiperazine peroxide, 13-*O*-prenyl-26-hydroxyverruculogen (**91**), was isolated and identified from the culture extract of the marine sediment-derived fungus *Penicillium brefeldianum* SD-273. Compound **91** showed potent lethality against brine shrimp (*Artemia salina*), with LD_50_ value of 9.44 μΜ, comparing with the positive control colchicine (LD_50_ = 99.0 μΜ) (Figure 20) [54].

**Figure 21:**
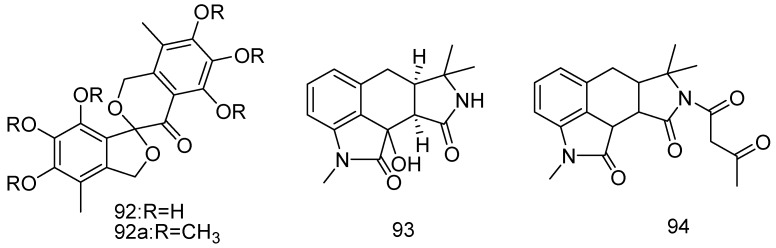
Structures of compounds **92**–**94**.

Fungus *Spicaria elegans* KLA03 was derived from marine sediments collected in Jiaozhou Bay, China. Eleganketal A (**92**), a naturally occurring aromatic polyketide possessing a rare highly oxygenated spiro[isobenzofuran-1,3′-isochroman] ring system, was isolated from the fungus by culturing it in a modified mannitol-based medium. The synthetic (±)-**92**a and its separated enantiomers showed no cytotoxicity against HL-60 and K562 cells (IC_50_ > 50 μM). Only compound (−)-**92**a exhibited activity against the influenza A H1N1 virus with an IC_50_ = 149 μM (Figure 21) [55].

Two novel tetracyclic oxindole alkaloids, speradines G (**93**) and H (**94**), were isolated from the marine-derived fungus *Aspergillus oryzae*, isolated from marine sediments (Langqi Island, Fujian, China). This is the first report on cyclopiazonic acid (CPA)-type alkaloids with a hexacyclic skeleton. The compounds **93**–**94** showed unconspicuous cytotoxic effects on the Hela, HL-60 and K562 cell lines, IC_50_ values larger than 30 μg/mL (Figure 21) [56].

**Figure 22:**
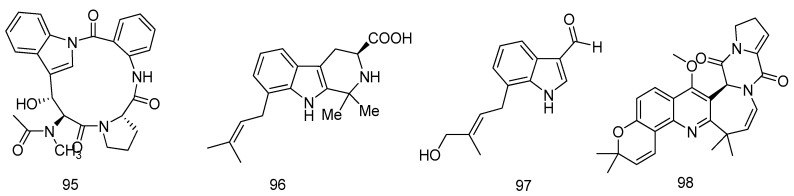
Structures of compounds **95**–**98**.

One new cyclic peptide, psychrophilins (**95**), possessing a rare amide linkage between the carboxylic acid in anthranilic acid (ATA) and the nitrogen from an indole moiety, was obtained from the culture of the marine-derived fungus *Aspergillus versicolor* ZLN-60, isolated from the mud (depth, 20 m) of the Yellow Sea. Compound **95** showed potent lipid-lowering effects at a dose of 10 μM as assessed by Oil Red O staining (Figure 22) [57].

Two new prenylated indole alkaloids, including a *β*-carboline, penipalines B (**96**), and one indole carbaldehyde derivative, penipaline C (**97**), were obtained from the deep-sea-sediment derived fungus *Penicillium paneum* SD-44 cultured in a 500-L bioreactor. Compounds **96** and **97** showed potent cytotoxic activities against two tumor cell lines, A-549 and HCT-116. The IC_50_ values of compounds **96** and **97** against HCT-116 were 14.88 and 18.54 μM, while those against A-549 were 20.44 and 21.54 μM, respectively (Figure 22) [58].

The fungal strain *Aspergillus versicolor* HDN08-60, isolated from the sediments in the South China Sea, was fermented on liquid culture (60 L) for 30 days and extracted three times with EtOAc. A novel versicamide H (**98**) was obtained. Compound **98** exhibited moderate activity against HL-60 cells (IC_50_ = 8.7 μM) and selective PTK inhibitory activities in further investigation with target screening (Figure 22) [59].

**Figure 23:**
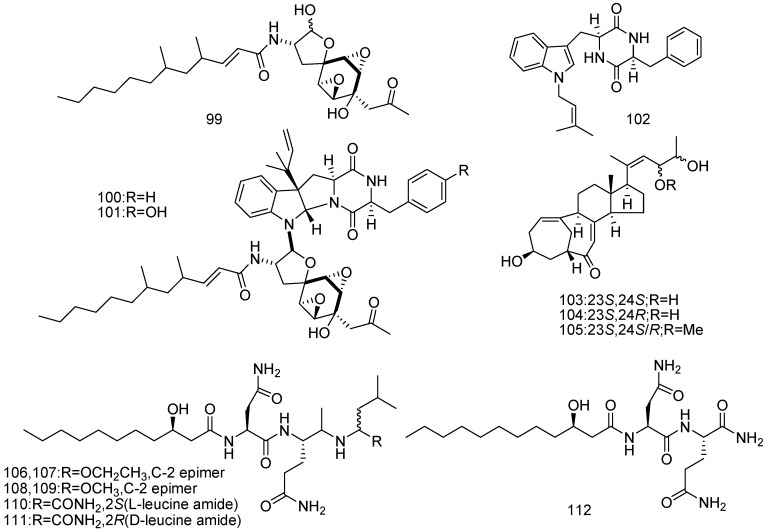
Structures of compounds **99**–**112**.

After modified diethyl sulphate mutagenesis procedure, a marine-derived fungus *Penicillium purpurogenum* G59 (the tideland of Bohai Bay, Tianjin, China) yielded four new antitumor compounds named penicimutanolone (**99**), penicimutanin A (**100**), penicimutanin B (**101**), and penicimutatin (**102**). Compounds **99**–**101** inhibited several human cancer cell lines (K562, HL-60, HeLa, BGC-823, and MCF-7) with IC_50_ values lower than 20 μM, compound **102** also inhibited the cell lines to some extent [60]. In addition, three new C25 steroids (**103**–**105**) with an unusual bicyclo[4.4.1]A/B ring with the *Z*-configuration of 20,22-double bond were isolated. All of them weakly inhibited several human cancer cell lines (K562, HL-60 and HeLa) to varying extents (Figure 23) [61]. Furthermore, seven new (**106**–**112**) lipopeptides were isolated from the extract of mutant, which showed weak cytotoxicity (Figure 23) [62]. These results provided the way to discover new compounds by activating silent fungal metabolic pathways.

**Figure 24:**
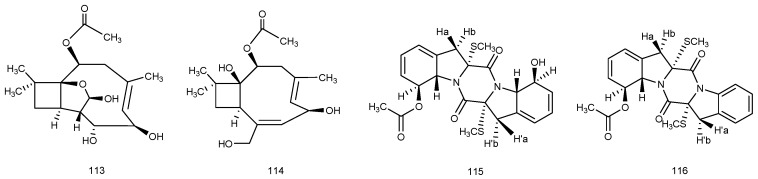
Structures of compounds **113**–**116**.

*Ascotricha* sp. ZJ-M-5, is a fungus isolated from a mud sample, which was collected on a coastal beach in Fenghua County, Zhejiang Province, China. Chemical investigations were found to produce cyclonerodiol analogues, a 3,4-*seco* lanostane triterpenoid, and diketopiperazines in an eutrophic medium by the one strain-many compounds (OSMAC) analysis. Two new caryophyllene derivatives (**113**–**114**) were produced in an oligotrophic medium, Czapek Dox broth with or without Mg^2+^. (+)-6-*O*-Demethylpestalotiopsin A (**113**) and (+)-6-*O*-demethylpestalotiopsin C (**114**), which have a five-membered hemiacetal structural moiety, showed growth inhibitory abilities against K562 and HL-60 leukemia cell lines with the lowest GI_50_ value of 6.9 ± 0.4 μM. This indicated that modification of the culture media was effective in the discovery of novel bioactive fungal secondary metabolites (Figure 24) [63].

The marine fungus *Cladosporium* sp. was isolated from a sediment sample collected from Yangshashan Bay, Ningbo, Zhejiang Province, China. Two new sulfur-containing diketopiperazines (DKPs), cladosporin A (**115**) and cladosporin B (**116**), were separated from the fungus by high-speed counter-current chromatography (HSCCC). Cytotoxic activity tests showed that compounds **115** and **116** exhibited moderate cytotoxic activities to HepG2 cell line, with values of IC_50_ 21 and 42 μg/mL (Figure 24) [64].

**Figure 25:**
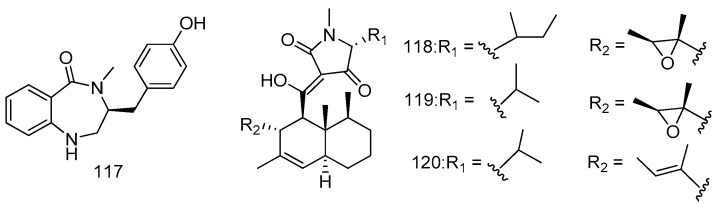
Structures of compounds **117**–**120**.

A novel cyclic dipeptide, 14-hydroxy-cyclopeptine (**117**), was purified from a deep sea derived fungus SCSIOW2 identified as an *Aspergillus* sp. Fungus SCSIOW2 was isolated from deep marine sediment sample collected in the South China Sea at a depth of 2439 m. Compound **117** inhibited nitric oxide production with IC_50_ value at 40.3 μg/mL in a lipopolysaccharide and recombinant mouse interferon-γ-activated macrophage-like cell line, RAW 264.7 (Figure 25) [65].

Trichobotrysins (**118**–**120**), a class of new tetramic acid derivatives with a decalin ring, were characterized from the culture of *Trichobotrys effuse* DFFSCS021 derived from the deep sea sediment collected from the South China Sea. Compounds **118**–**120** exhibited significant selective cytotoxicity against human carcinoma KG-1a cell line with IC_50_ values of 5.44, 8.97, and 6.16 μM, and obvious antiviral activity towards HSV-1 with IC_50_ values of 3.08, 9.37, and 3.12 μM, respectively (Figure 25) [66].

### 2.4. Alga

**Figure 26:**
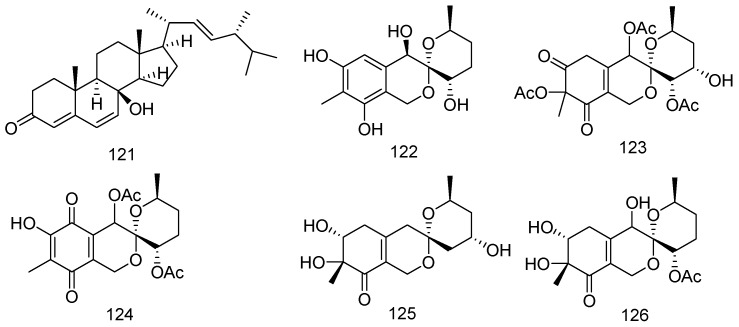
Structures of compounds **121**–**126**.

The *Aspergillus ustus* cf-42 strain, which was obtained from the fresh tissue of the marine green alga *C. fragile* (Zhoushan Island, China), generated a new ergosteroid derivative, isocyathisterol (**121**). Compound **121** exhibited weak antibacterial activity against *S. aureus* and *E. coli* (inhibitory diameters of 5.7 and 6.7 mm, respectively) at 30 mg/disc (Figure 26) [67].

Five new polyketides (**122**–**126**) have been isolated from the lipophilic extracts of the marine-derived fungi *Penicillium thomii* and *Penicillium lividum* isolated from superficial mycobiota of the brown alga *Sargassum miyabei* (Lazurnaya Bay, the Sea of Japan). Compound **123** was able to inhibit the transcriptional activity of the oncogenic nuclear factor AP-1 with IC_50_ value of 15 μM after 12 h of treatment. Compound **125** exhibited cytotoxicity against splenocytes with a IC_50_ value of 38 μM. It was shown that compounds **124** and **126** at a non-toxic concentration (10 μM) inhibited the adhesion of macrophages (30%–40% of inhibition). In addition, compounds **122** and **125** exhibited radical scavenging activity against DPPH with IC_50_ values of 100 and 50 μM, respectively (Figure 26) [68].

**Figure 27:**
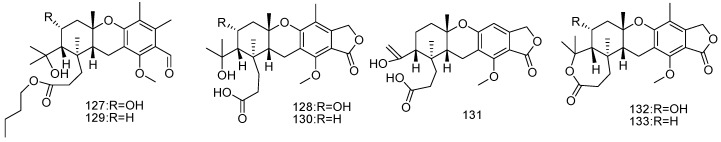
Structures of compounds **127**–**133**.

Seven new austalide meroterpenoids (**127**–**133**) were isolated from the alga-derived fungi *Penicillium thomii* KMM 4645 and *Penicillium lividum* KMM 4663, which was isolated from superficial mycobiota of the brown alga *Sargassum miyabei* (Lazurnaya Bay, the Sea of Japan). Compounds **127**, **128**, **132** and **133** could inhibit AP-1-dependent transcriptional activity in JB6 Cl41 cell lines at noncytotoxic concentrations. Compounds **127**–**133** exhibited significant inhibitory effects against endo-1,3-β-d-glucanase from a crystalline stalk of the marine mollusk *Pseudocardium sachalinensis* (Figure 27) [69].

**Figure 28:**
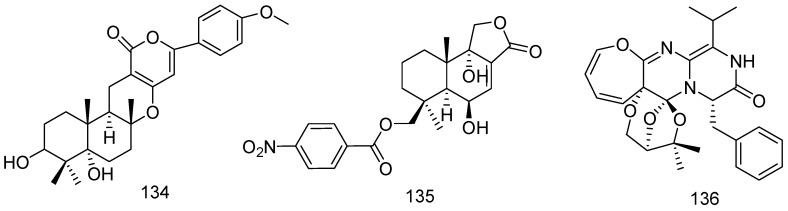
Structures of compounds **134**–**136**.

The fungal strain *Penicillium echinulatum* pt-4 was isolated from marine red alga *Chondrus ocellatus* that was collected from the coast of Pingtan Island, China. One new meroterpene, arisugacin K (**134**) was isolated from the culture of strain pt-4. Compound **134** showed inhibitory activity against *E. coli* with an inhibition diameter 8 mm at 30 μg/disk (Figure 28) [70].

A new nitrobenzoyl sesquiterpenoid, 6b,9a-dihydroxy-14-*p*-nitrobenzoylcinnamolide (**135**) was isolated from extracts of the culture of marine-derived fungus *Aspergillus ochraceus* Jcma1F17, which was derived from a marine alga *Coelarthrum* sp. in Paracel Islands, South China Sea. Compound **135** displayed significant cytotoxicities against 10 cancer cell lines (K562, H1975, U937, Molt-4, BGC-823, HL60, MCF-7, A549, Hela, and Huh-7), with IC_50_ values of 1.95 μM to 6.35 μM. In addition, compound **135** also showed antiviral activities against EV71 and H3N2 (Figure 28) [71].

A structurally unique 3*H*-oxepine-containing alkaloid, varioxepine A (**136**), characterized by a condensed 3,6,8-trioxabicyclo[3.2.1]octane motif, was isolated from the marine algal-derived endophytic fungus *Paecilomyces variotii*. Compound **136** was evaluated for antimicrobial activity against several human- and aqua-pathogenic bacteria (*Aeromonas hydrophila*, *S. aureus*, *Vibrio anguillarum*, *E. coli*, *Micrococcus luteus*, *Vibrio harveyi*, and *Vibrio parahemolyticus*). The results revealed that compound **136** has diverse antibacterial activities with the MIC values ranging from 16 to 64 μg/mL. Furthermore, it inhibited plant pathogenic fungus *Fusarium graminearum*, with an MIC value of 4 μg/mL (Figure 28) [72].

**Figure 29:**
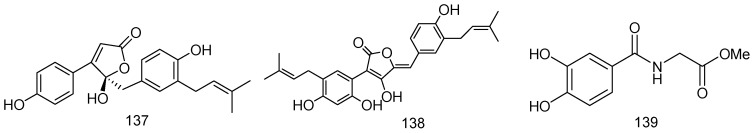
Structures of compounds **137**–**139**.

Two new butenolides, namely, butyrolactone IX (**137**) and aspulvinone O (**138**) were isolated from the marine-derived endophytic fungus *Paecilomyces variotii* from *Grateloupia turuturu*, a red alga collected from the coast of Qingdao, China. The isolated butenolides were tested for the activity against DPPH radicals and the results indicated that butyrolactone (**137**) possessed potent activity with IC_50_ values 186.3 μM, while aspulvinone (**138**) showed significant activity with IC_50_ value 11.6 μM. The author speculated that a larger conjugated aromatic system gave aspulvinone (**138**) more stronger DPPH radical scavenging activity than that of butyrolactone (**137**) (Figure 29) [73].

A new benzamide derivative (methyl 4-(3,4-dihydroxybenzamido) butanoate (**139**) was isolated from themarine brown alga-derived endophytic fungus *Aspergillus wentii* EN-48. Compound **139** showed significant scavenging activity against DPPH with IC_50_ values of 5.2 μg/mL, which was significantly stronger than BHT (IC_50_ = 36.9 μg/mL) (Figure 29) [74].

**Figure 30:**
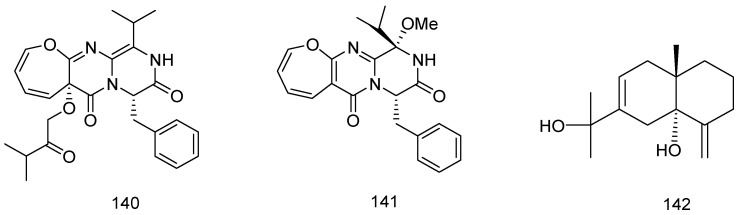
Structures of compounds **140**–**142**.

Two new oxepine-containing diketopiperazine-type alkaloids, varioloids A and B (**140** and **141**), were isolated from the fungus *Paecilomyces variotii* EN-291, which was isolated from *Grateloupia turuturu*, a marine red algae collected from the coast of Qingdao, China. Compounds **140** and **141** exhibited potent activity against the plantpathogenic fungus *Fusarium graminearum* with *MIC* values of 8 and 4 μg/mL, respectively (Figure 30) [75].

A new eudesmane sesquiterpenoid, eudesma-4(15),7-diene-5,11-diol (**142**) has been isolated from the red alga *Laurencia obtusa*, which was collected off the Saudi Arabia Red Sea Coast at Jeddah. Both qualitative and quantitative antifungal assays revealed that compound **142** exhibited a good antifungal effect against *Candida albicans*, *Candida tropicals*, *Aspergillus flavus* and *Aspergillus niger*; the *MIC* values were 2.92, 2.10, 2.92, 6.5 μg/mL, respectively (Figure 30) [76].

### 2.5. Sea Water

**Figure 31:**
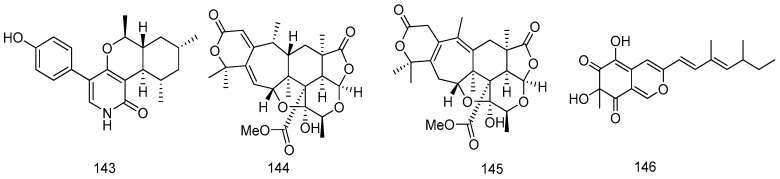
Structures of compounds **143**–**146**.

One unusual pyridone, trichodin A (**143**), was extracted from the marine fungus, *Trichoderma* sp. strain MF106 isolated from the Greenland Seas. Compound **143** showed antibiotic activities against *Staphylococcus epidermidis* with IC_50_ value of 24 μM (Figure 31) [77].

The fungus *Penicillium* 303# was isolated from sea water, which was collected from Zhanjiang Mangrove National Nature Reserve in Guangdong Province, China. Three new metabolites (compounds **144**–**146**) were isolated from the fungus fermentation medium. Those compounds showed weak to moderate cytotoxic activities against MDA-MB-435 (Figure 31) [78].

**Figure 32:**
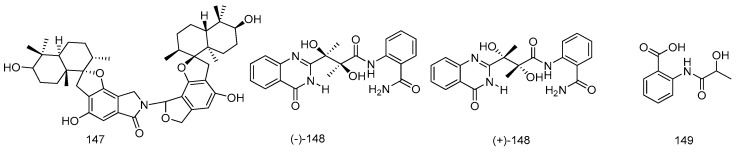
Structures of compounds **147**–**149**.

A marine strain *Stachybotrys* sp. MF347, which was isolated from a driftwood sample collected at Helgoland (North Sea, Germany), provided a novel spirocyclic drimane coupled by two drimane fragment building blocks **147**. Compound **147** exhibited comparable antibacterial activities with chloramphenicol against the clinically relevant MRSA (Figure 32) [79].

Penicilliumine (**148**), a new structure was isolated from the fermentation *Penicillium* *commune* 366606, a marine-derived fungus isolated from the sea water collected at Qingdao, China. Compound **148** was not cytotoxic against MCF-7, SMMC-7721, HL-60, A-549 and SW480 cells or no potent inhibiting the nitric oxide release. Compound (−)-**148** and (+)-**148** could inhibit the acetylcholinesterase activity by 18.7% (±0.26%) and 32.4% (±2.08%) at the concentration of 50 μM, respectively, compared with 43.6% (±2.12%) inhibition rate of the positive control tacrine (Figure 32) [80].

A strain of the fungus *Penicillium chrysogenum* was collected from sea water (10–25 m depth), off the North Sea coast, China. A new benzoic acid, 2-(2-hydroxypropanamido) benzoic acid (**149**), isolated from the fermentation broth of fungus, showed remarkable anti-inflammatory and analgesic activities but exhibited no ulcerogenic effect (Figure 32) [81].

### 2.6. Others

**Figure 33:**
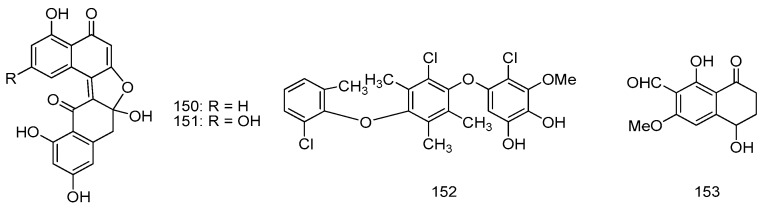
Structures of compounds **150**–**153**.

Racemic dinaphthalenone derivatives (±)-asperlone A (**150**) and (±)-asperlone B (**151**) were isolated from the cultures of *Aspergillus* sp. 16-5C from the leaves of *S. apetala*, which were collected in Hainan Island, China. Compounds **150** and **151** exhibited potent inhibitory effects against *Mycobacterium tuberculosis* protein tyrosine phosphatase B (MptpB) with IC_50_ values of 4.24 ± 0.41, 4.32 ± 0.60 μM, respectively, which represent a new type of lead compounds for the development of new anti-tuberculosis drugs (Figure 33) [82].

A new polychlorinated triphenyl diether named microsphaerol (**152**) and a new naphthalene derivative named seimatorone (**153**), were isolated from the endophtic fungus *Microsphaeropsis* sp. and *Seimatosporium* sp., which were isolated from the halotolerant herbaceous plant *Salsola oppositifolia* from Playa del Ingles (Gomera, Spain). Preliminary studies revealed that compound **152** showed good antibacterial activities against *Bacillus* *Megaterium* and *E*. *coli*, and good antilagal and antifungal activities against *Chlorella* *fusca* and *Microbotryum* *violaceum*, respectively. On the other hand, compound **153** exhibited moderate antibacterial, antialgal, and antifungal activities (Figure 33) [83].

## 3. Future Perspectives and Concluding Remarks

**Figure 34:**
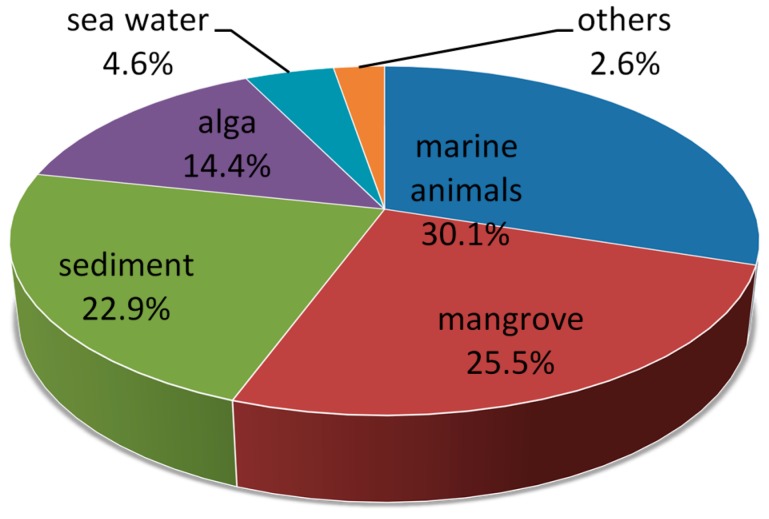
New compounds from marine-derived fungi included in this review, divided by sources of the fungal strains.

Based on the above literature, we can find that marine-derived *Aspergillus* and *Penicillium* are the most ubiquitous genera, probably because both of them are salt tolerant, fast growing and easily obtained. As seen in Figure 34, about 3/4 of all new compounds reported from marine fungi are derived from isolation from living matter, *i.e.* marine animals (30.1%) and marine plants (42.5%), while the remaining compounds are obtained from non-living sources, most notably sediments (22.9%). Within the individual groups, mangrove habitats (25.5%), alga (14.4%), and sponges (9.2%) are the predominant sources for fungal diversity. A newly emerging source is the deep sea. The extreme environment encountered in the form of low temperature, elevated hydrostatic pressure, absence of light, high concentrations of metals in hydrothermal vents and hypoxic conditions possibly produce structurally unique metabolites. Nevertheless, very few reports are related to this habitat because of scarcity of source. It is worth mentioning that an increasing number of Chinese scientists are engaging in this research field, mostly focusing on mangrove areas around South China Sea.

**Figure 35:**
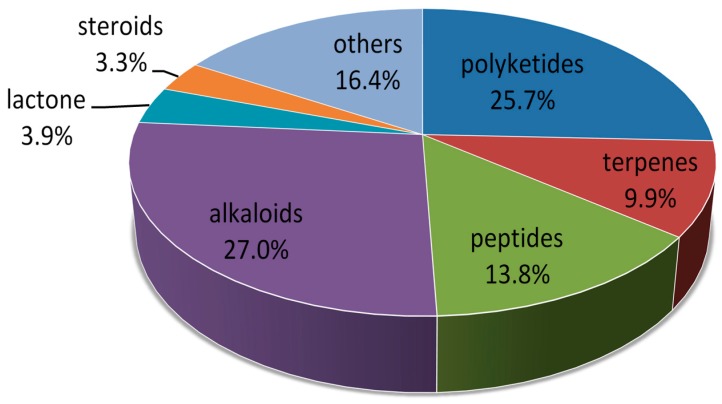
New compounds from marine-derived fungi included in this review, divided by structural types.

According to the structural types, of the 153 compounds included in this review, alkaloids (27.0%) and polyketides (25.7%) play a dominant role. Moreover, peptides, terpenes, lactone, and steroids are 13.8%, 9.9%, 3.9% and 3.3%, respectively (see Figure 35).

**Figure 36:**
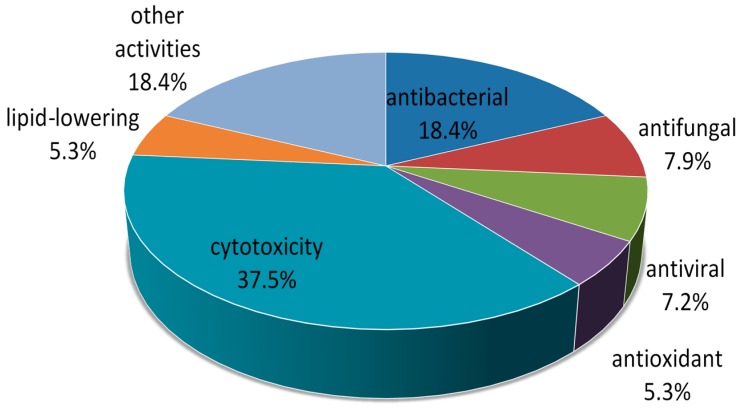
Bioactive categories of new compounds from marine-derived fungi included in this review.

As illustrated in Figure 36, biological activities of these compounds are mainly focused in the areas of cytotoxicity (37.5%) and antimicrobial activity, including antibacterial activity (18.4%), antifungal activity (7.9%) and antiviral activity (7.2%). Furthermore, other selective activities include antioxidant, anti-inflammatory, antifouling, lipid-lowering activities, lethality against brine shrimp effects, *etc.*

The oceans are the largest underexploited wealthy resource of potential drugs. Marine-derived fungi have provided a variety of potential pharmacological metabolites and thus represent a valuable resource of new drug candidates. In the period covered by the first review of this series, from the beginning until 2002, 272 new structures had been reported, in 2009 more than 200 was reached [2], and in 2012 and 2013, the numbers were 288 and 302, respectively [3,84]. Though bioactivities of secondary metabolites from marine fungi reveal interesting levels for a number of clinical relevant targets, they are not well represented in the pipelines of drugs and none of them currently is on the market. Only Plinabulin, a synthetic cyclic dipeptide analogue of halimide, which is isolated from a marine fungus species, is in phase II clinical trial for treatment of non-small cell lung cancer. Thus, there is still a long way to go [85].

First of all, many marine-derived fungal biosynthetic pathways are silent under common laboratory culture conditions, and activation of the silent pathways may enable access to new metabolites. One strain–many compounds (OSMAC) strategy, chemical epigenetic modification (e.g., using DNA methyltransferase inhibitor, 5-azacytidine, histone deacetylase inhibitors, suberoylanilide hydroxamic acid and sodium butyrate [60,61,62,86,87,88]), co-culture method [48], or gene level manipulations could be applied to access new secondary metabolites. Furthermore, as mentioned above, alterations of the culture conditions might lead to changes of the metabolic spectrum. The pharmaceutical industry should concentrate on how to appropriately maintain certain physico-chemical factors, *viz*., amount of oxygen available, optimum pH and temperature, avoiding variation of secondary metabolites.

What is more, a better understanding of the molecular basis of biosynthesis and regulation mechanisms will contribute to making better use of the enormous chemical potential of marine derived fungi, which depends on the continuous development of the new techniques [89,90].

In addition, beyond the current *in vitro* bioactivity examination, further *in vivo* and preclinical studies, as well as side effects examinations, are required to determine the bioactive compounds with potential therapeutic applications.

We believe that with the development of more automated and more affordable techniques for isolating and characterizing marine fungi bioactive metabolites, marine fungi will be promising sources for novel therapeutic agents that will be useful in controlling human diseases and protecting human health.

## References

[1] Schueffler A., Anke T. (2014). Fungal natural products in research and development. Nat. Prod. Rep..

[2] Rateb M.E., Ebel R. (2011). Secondary metabolites of fungi from marine habitats. Nat. Prod. Rep..

[3] Blunt J.W., Copp B.R., Keyzers R.A., Munro M.H.G., Prinsep M.R. (2015). Marine natural products. Nat. Prod. Rep..

[4] Hamilton-Miller J.M.T. (2008). Development of the semi-synthetic penicillins and cephalosporins. Int. J. Antimicrob. Agents.

[5] Blunt J.W., Copp B.R., Munro M.H.G., Northcote P.T., Prinsep M.R. (2011). Marine natural products. Nat. Prod. Rep..

[6] Kohlmeyer J., Kohlmeyer E. (1979). Marine Mycology: The Higher Fungi.

[7] Wang J., Wei X., Qin X., Lin X., Zhou X., Liao S., Yang B., Liu J., Tu C., Liu H. (2015). Arthpyrones A–C, pyridone alkaloids from a sponge-derived fungus *Arthrinium arundinis* ZSDS1-F3. Org. Lett..

[8] Li Y., Wu C., Liu D., Proksch P., Guo P., Lin W.H. (2014). Chartarlactams A–P, phenylspirodrimanes from the sponge-associated fungus *Stachybotrys chartarum* with antihyperlipidemic activities. J. Nat. Prod..

[9] Song F., Ren B., Chen C., Yu K., Liu X., Zhang Y., Yang N., He H., Liu X., Dai H., Zhang L. (2014). Three new sterigmatocystin analogues from marine-derived fungus *Aspergillus versicolor* MF359. Appl. Microbiol. Biotechnol..

[10] Khamthong N., Rukachaisirikul V., Phongpaichit S., Preedanon S., Sakayaroj J. (2014). An antibacterial cytochalasin derivative from the marine-derived fungus *Diaporthaceae* sp. PSU-SP2/4. Phytochem. Lett..

[11] Prompanya C., Dethoup T., Bessa L.J., Pinto M.M.M., Gales L., Costa P.M., Silva A.M.S., Kijjoa A. (2014). New isocoumarin derivatives and meroterpenoids from the marine sponge-associated fungus *Aspergillus similanensis* sp. Nov. KUFA 0013. Mar. Drugs.

[12] Kotoku N., Higashimoto K., Kurioka M., Arai M., Fukuda A., Sumii Y., Sowa Y., Sakai T., Kobayashi M. (2014). Xylarianaphthol-1, a novel dinaphthofuran derivative, activates p21 promoter in a p53-independent manner. Bioorg. Med. Chem. Lett..

[13] Wu B., Wiese J., Labes A., Kramer A., Schmaljohann R., Imhoff J.F. (2015). Lindgomycin, an unusual antibiotic polyketide from a marine fungus of the Lindgomycetaceae. Mar. Drugs.

[14] Nong X., Wang Y., Zhang X., Zhou M., Xu X., Qi S. (2014). Territrem and butyrolactone derivatives from a marine-derived fungus *Aspergillus terreus*. Mar. Drugs.

[15] Bao J., Luo J., Qin X., Xu X., Zhang X., Tu Z., Qi S. (2014). Dihydrothiophene-condensed chromones from a marine-derived fungus *Penicillium oxalicum* and their structure-bioactivity relationship. Bioorg. Med. Chem. Lett..

[16] Dong J., Bao J., Zhang X., Xu X., Nong X., Qi S. (2014). Alkaloids and citrinins from marine-derived fungus *Nigrospora oryzae* SCSGAF 0111. Tetrahedron Lett..

[17] Chen M., Fu X., Kong C., Wang C. (2014). Nucleoside derivatives from the marine-derived fungus *Aspergillus versicolor*. Nat. Prod. Res..

[18] Liu Z., Xia G., Chen S., Liu Y., Li H., She Z. (2014). Eurothiocin A and B, sulfur-containing benzofurans from a soft coral-derived fungus *Eurotium rubrum* SH-823. Mar. Drugs.

[19] Li H., Jiang W., Liang W., Huang J., Mo Y., Ding Y., Lam C., Qian X., Zhu X., Lan W. (2014). Induced marine fungus *Chondrostereum* sp. as a means of producing new sesquiterpenoids chondrosterins I and J by using glycerol as the carbon source. Mar. Drugs.

[20] Chen M., Wang K., Liu M., She Z., Wang C. (2015). Bioactive steroid derivatives and butyrolactone derivatives from a gorgonian-derived *Aspergillus* sp. fungus. Chem. Biodivers..

[21] Chen M., Han L., Shao C., She Z., Wang C. (2015). Bioactive diphenyl ether derivatives from a gorgonian-derived fungus *Talaromyces* sp.. Chem. Biodivers..

[22] Liang W., Le X., Li H., Yang X., Chen J., Xu J., Liu H., Wang L., Wang K., Hu K. (2014). Exploring the chemodiversity and biological activities of the secondary metabolites from the marine fungus *Neosartorya Pseudofischeri*. Mar. Drugs.

[23] Lan W., Liu W., Liang W., Xu Z., Le X., Xu J., Lam C.K., Yang D., Li H., Wang L. (2014). Pseudaboydins A and B: Novel isobenzofuranone derivatives from marine fungus *Pseudallescheria boydii* associated with starfish *Acanthaster planci*. Mar. Drugs.

[24] Du F., Zhang P., Li X., Li C., Cui C., Wang B. (2014). Cyclohexadepsipeptides of the isaridin class from the marine-derived fungus *Beauveria felina* EN-135. J. Nat. Prod..

[25] Du F., Li X., Zhang P., Li C., Wang B. (2014). Cyclodepsipeptides and other o-containing heterocyclic metabolites from *Beauveria felina* EN-135, a marine-derived entomopathogenic fungus. Mar. Drugs.

[26] Quang T.H., Ngan T.T.N., Ko W., Kim D.C., Yoon S.C., Sohn J.H., Yim J.H., Kim Y.C., Oh H. (2014). Tanzawaic acid derivatives from a marine isolate of *Penicillium* sp. (SF-6013) with anti-inflammatory and PTP1B inhibitory activities. Bioorg. Med. Chem. Lett..

[27] Zhu T., Chen Z., Liu P., Wang Y., Xin Z., Zhu W. (2014). New rubrolides from the marine-derived fungus *Aspergillus Terreus* OUCMDZ-1925. J. Antibiot..

[28] Sun K., Li Y., Guo L., Wang Y., Liu P., Zhu W. (2014). Indole diterpenoids and isocoumarin from the fungus, *Aspergillus flavus*, isolated from the prawn, *Penaeus vannamei*. Mar. Drugs.

[29] Yang X., Kang M., Li Y., Kim E.A., Kang S., Jeon Y.J. (2014). Anti-inflammatory activity of questinol isolated from marine-derived fungus *Eurotium amstelodami* in lipopolysaccharide-stimulated RAW 264.7 macrophages. J. Microbiol. Biotechnol..

[30] Liu Y., Zhao S., Ding W., Wang P., Yang X., Xu J. (2014). Methylthio-aspochalasins from a marine-derived fungus *Aspergillus* sp.. Mar. Drugs.

[31] Murshid S.S.A., Badr J.M., Youssef D.T.A. (2016). Penicillosides A and B: New cerebrosides from the marine-derived fungus *Penicillium* species. Rev. Bras. Farmacogn..

[32] Ma Y., Li J., Huang M., Liu L., Wang J., Lin Y. (2015). Six new polyketide decalin compounds from mangrove endophytic fungus *Penicillium aurantiogriseum* 328#. Mar. Drugs.

[33] Liu Y., Chen S., Liu Z., Lu Y., Xia G., Liu H., He L., She Z. (2015). Bioactive metabolites from mangrove endophytic fungus *Aspergillus* sp. 16–5B. Mar. Drugs.

[34] Zhang P., Meng L., Mandi A., Kurtan T., Li X., Liu Y., Li X., Li C., Wang B. (2014). Brocaeloids A–C, 4-oxoquinoline and indole alkaloids with C-2 reversed prenylation from the mangrove-derived endophytic fungus *Penicillium brocae*. Eur. J. Org. Chem..

[35] Kong F., Wang Y., Liu P., Dong T., Zhu W. (2014). Thiodiketopiperazines from the marine-derived fungus *Phoma* sp. OUCMDZ-1847. J. Nat. Prod..

[36] Meng L., Li X., Lu C., Huang C., Wang B. (2014). Brocazines A–F, cytotoxic bisthiodiketopiperazine derivatives from *Penicillium brocae* MA-231, an endo-phytic fungus derived from the marine mangrove plant *Avicennia marina*. J. Nat. Prod..

[37] Yang J., Qiu S., She Z., Lin Y. (2014). A new isochroman derivative from the marine fungus *Phomopsis* sp. (No.Gx-4). Chem. Nat. Compd..

[38] Meng L., Zhang P., Li X., Wang B. (2015). Penicibrocazines A–E, five new sulfide diketopiperazines from the marine-derived endophytic fungus *Penicillium brocae*. Mar. Drugs.

[39] Li H., Jiang J., Liu Z., Lin S., Xia G., Xia X., Ding B., He L., Lu Y., She Z. (2014). Peniphenones A–D from the mangrove fungus *Penicillium dipodomyicola* HN4-3A as inhibitors of *Mycobacterium tuberculosis* Phosphatase MptpB. J. Nat. Prod..

[40] Luo H., Li X., Li C., Wang B. (2014). Diphenyl ether and benzophenone derivatives from the marine mangrove-derived fungus *Penicillium* sp. MA-37. Phytochem. Lett..

[41] Wang J., Wei X., Lu X., Xu F., Wan J., Lin X., Zhou X., Liao S., Yang B., Tu Z., Liu Y. (2014). Eight new polyketide metabolites from the fungus *Pestalotiopsis vaccinii* endogenous with the mangrove plant *Kandelia candel* (L.) *Druce*. Tetrahedron.

[42] Zhou X., Lin X., Ma W., Fang W., Chen Z., Yang B., Liu Y. (2014). A new aromatic amine from fungus *Pestalotiopsis vaccinii*. Phytochem. Lett..

[43] Kornsakulkarn J., Saepua S., Komwijit S., Rachtawee P., Thongpanchang C. (2014). Bioactive polyketides from the fungus *Astrocystis* sp. BCC 22166. Tetrahedron.

[44] Bai Z., Lin X., Wang Y., Wang J., Zhou X., Yang B., Liu J., Yang X., Wang Y., Liu Y. (2014). New phenyl derivatives from endophytic fungus *Aspergillus flavipes* AIL8 derived of mangrove plant *Acanthus ilicifolius*. Fitoterapia.

[45] Wang J., Wei X., Qin X., Chen P., Lin X., Zhang T., Yang X., Liao S., Yang B., Liu J., Zhou X., Tu Z., Liu Y. (2015). Two new prenylated phenols from endogenous fungus *Pestalotiopsis vaccinii* of mangrove plant *Kandelia candel* (L.) *Druce*. Phytochem. Lett..

[46] Meng L., Li X., Liu Y., Wang B. (2014). Penicibilaenes A and B, sesquiterpenes with a tricyclo[6.3.1.0(1,5)]dodecane skeleton from the marine isolate of *Penicillium bilaiae* MA-267. Org. Lett..

[47] Wang J., Cox D.G., Ding W., Huang G., Lin Y., Li C. (2014). Three new resveratrol derivatives from the mangrove endophytic fungus *Alternaria* sp.. Mar. Drugs.

[48] Huang S., Ding W., Li C., Cox D.G. (2014). Two new cyclopeptides from the co-culture broth of two marine mangrove fungi and their antifungal activity. Pharmacogn. Mag..

[49] Li C., Wang J., Luo C., Ding W., Cox D.G. (2014). A new cyclopeptide with antifungal activity from the co-culture broth of two marine mangrove fungi. Nat. Prod. Res..

[50] Peng J., Zhang X., Du L., Wang W., Zhu T., Cu Q., Li D. (2014). Sorbicatechols A and B, antiviral sorbicillinoids from the marine-derived fungus *Penicillium chrysogenum* PJX-17. J. Nat. Prod..

[51] Liao L., Lee J.H., You M.J., Choi T.J., Park W., Lee S.K., Oh D.C., Oh K.B., Shin J. (2014). Penicillipyrones A and B, meroterpenoids from a marine-derived *Penicillium* sp. fungus. J. Nat. Prod..

[52] Orfali R.S., Aly A.H., Ebrahim W., Abdel-Aziz M.S., Müller W.E.G., Lin W.H., Daletos G., Proksch P. (2015). Pretrichodermamide C and N-methylpretrichodermamide B, two new cytotoxic epidithiodiketopiperazines from hyper saline lake derived *Penicillium* sp.. Phytochem. Lett..

[53] Yurchenko A.N., Smetanina O.F., Kalinovsky A.I., Pushilin M.A., Glazunov V.P., Khudyakova Y.V., Kirichuk N.N., Ermakova S.P., Dyshlovoy S.A., Yurchenko E.A. (2014). Oxirapentyns F–K from the marine-sediment-derived fungus *Isaria felina* KMM 4639. J. Nat. Prod..

[54] An C., Li X., Li C., Xu G., Wang B. (2014). Prenylated indolediketopiperazine peroxides and related homologues from the marine sediment-derived fungus *Penicillium brefeldianum* SD-273. Mar. Drugs.

[55] Luan Y., Wei H., Zhang Z., Che Q., Liu Y., Zhu T., Mandi A., Kurtan T., Gu Q., Li D. (2014). Eleganketal A, a highly oxygenated dibenzospiroketal from the marine-derived fungus *Spicaria elegans* KLA03. J. Nat. Prod..

[56] Hu X., Xia Q., Zhao Y., Zheng Q., Liu Q., Chen L., Zhang Q. (2014). Speradines F–H, three new oxindole alkaloids from the marine-derived fungus *Aspergillus oryzae*. Chem. Pharm. Bull..

[57] Peng J., Gao H., Zhang X., Wang S., Wu C., Gu Q., Guo P., Zhu T., Li D. (2014). Psychrophilins E–H and versicotide C, cyclic peptides from the marine-derived fungus *Aspergillus versicolor* ZLN-60. J. Nat. Prod..

[58] Li C., Li X., An C., Wang B. (2014). Prenylated indole alkaloid derivatives from marine sediment-derived fungus *Penicillium paneum* SD-44. Helvetica. Chim. Acta.

[59] Peng J., Gao H., Li J., Ai J., Geng M., Zhang G., Zhu T., Gu Q., Li D. (2014). Prenylated indole diketopiperazines from the marine-derived fungus *Aspergillus versicolor*. J. Org. Chem..

[60] Fang S., Wu C., Li C., Cui C. (2014). A practical strategy to discover new antitumor compounds by activating silent metabolite production in fungi by diethyl sulphate mutagenesis. Mar. Drugs.

[61] Xia M., Cui C., Li C., Wu C. (2014). Three new and eleven known unusual C25 steroids: Activated production of silent metabolites in a marine-derived fungus by chemical mutagenesis strategy using diethyl sulphate. Mar. Drugs.

[62] Wu C., Li C., Cui C. (2014). Seven new and two known lipopeptides as well as five known polyketides: The activated production of silent metabolites in a marine-derived fungus by chemical mutagenesis strategy using diethyl sulphate. Mar. Drugs.

[63] Wang W., Li D., Li Y., Hua H., Ma E., Li Z. (2014). Caryophyllene sesquiterpenes from the marine-derived fungus *Ascotricha* sp. ZJ-M-5 by the one strain-many compounds strategy. J. Nat. Prod..

[64] Gu B., Zhang Y., Ding L., He S., Wu B., Dong J., Zhu P., Chen J., Zhang J., Yan X. (2015). Preparative separation of sulfur-containing diketopiperazines from marine fungus *Cladosporium* sp. using high-speed counter-current chromatography in stepwise elution mode. Mar. Drugs.

[65] Zhou X., Fang P., Tang J., Wu Z., Li X., Li S., Wang Y., Liu G., He Z., Gou D. (2016). A novel cyclic dipeptide from deep marine-derived fungus *Aspergillus* sp. SCSIOW2. Nat. Prod. Res..

[66] Sun Y., Wang J., Wang Y., Zhang X., Nong X., Chen M., Xu X., Qi S. (2015). Cytotoxic and antiviral tetramic acid derivatives from the deep-sea-derived fungus *Trichobotrys effus* DFFSCS021. Tetrahedron.

[67] Liu X., Miao F., Liang X., Ji N. (2014). Ergosteroid derivatives from an algicolous strain of *Aspergillus ustus*. Nat. Prod. Res..

[68] Zhuravleva O.I., Sobolevskaya M.P., Afiyatullov S.S., Kirichuk N.N., Denisenko V.A., Dmitrenok P.S., Yurchenko E.A., Dyshlovoy S.A. (2014). Sargassopenillines A–G, 6,6-spiroketals from the alga-derived fungi *Penicillium thomii* and *Penicillium lividum*. Mar. Drugs.

[69] Zhuravleva O.I., Sobolevskaya M.P., Leshchenko E.V., Kirichuk N.N., Denisenko V.A., Dmitrenok P.S., Dyshlovoy S.A., Zakharenko A.M., Kim N.Y., Afiyatullov S.S. (2014). Meroterpenoids from the alga-derived fungi *Penicillium thomii* maire and *Penicillium lividum* westling. J. Nat. Prod..

[70] Li X., Miao F., Liang X., Ji N. (2014). Meroterpenes from an algicolous strain of *Penicillium echinulatum*. Magn. Reson. Chem..

[71] Fang W., Lin X., Zhou X., Wan J., Lu X., Yang B., Ai W., Lin J., Zhang T., Tu Z., Liu Y. (2014). Cytotoxic and antiviral nitrobenzoyl sesquiterpenoids from the marine-derived fungus *Aspergillus ochraceus* Jcma1F17. Med. Chem. Commun..

[72] Zhang P., Mandi A., Li X., Du F., Wang J., Li X., Kurtan T., Wang B. (2014). Varioxepine A, a 3H-oxepine-containing alkaloid with a new oxa-cage from the marine algal-derived endophytic fungus *Paecilomyces variotii*. Org. Lett..

[73] Zhang P., Li X., Wang J., Li X., Wang B. (2015). New butenolide derivatives from the marine-derived fungus *Paecilomyces variotii* with DPPH radical scavenging activity. Phytochem. Lett..

[74] Li X., Li X., Xu G., Li C., Wang B. (2014). Antioxidant metabolites from marine alga-derived fungus *Aspergillus wentii* EN-48. Phytochem. Lett..

[75] Zhang P., Li X., Wang J., Wang B. (2015). Oxepine-containing diketopiperazine alkaloids from the algal-derived endophytic fungus *Paecilomyces variotii* EN-291. Helv. Chim. Acta.

[76] Alarif W.M., Al-Footy K.O., Zubair M.S., Halid Ph M., Ghandourah M.A., Basaif S.A., Al-Lihaibi S.S., Ayyad S.N., Badria F.A. (2015). The role of new eudesmane-type sesquiterpenoid and known eudesmane derivatives from the red alga *Laurencia obtusa* as potential antifungal-antitumour agents. Nat. Prod. Res..

[77] Wu B., Oesker V., Wiese J., Schmaljohann R., Imhoff J.F. (2014). Two new antibiotic pyridones produced by a marine fungus, *Trichoderma* sp. strain MF106. Mar. Drugs.

[78] Li J., Yang X., Lin Y., Yuan J., Lu Y., Zhu X., Li J., Li M., Lin Y., He J., Liu L. (2014). Meroterpenes and azaphilones from marine mangrove endophytic fungus *Penicillium* 303#. Fitoterapia.

[79] Wu B., Oesker V., Wiese J., Malien S., Schmaljohann R., Imhoff J.F. (2014). Spirocyclic drimanes from the marine fungus *Stachybotrys* sp. strain MF347. Mar. Drugs.

[80] He J., Ji Y., Hu D., Zhang S., Yan H., Liu X., Luo H., Zhu H. (2014). Structure and absolute configuration of penicilliumine, a new alkaloid from *Penicillium commune* 366606. Tetrahedron Lett..

[81] Wang J., Zhao Y., Men L., Zhang Y., Liu Z., Sun T., Geng Y., Yu Z. (2014). Secondary metabolites of the marine fungus *Penicillium chrysogenum*. Chem. Nat. Compd..

[82] Xiao Z., Lin S., Tan C., Lu Y., He L., Huang X., She Z. (2015). Asperlones A and B, dinaphthalenone derivatives from a mangrove endophytic fungus *Aspergillus* sp. 16–5C. Mar. Drugs.

[83] Hussain H., Root N., Jabeen F., Al-Harrasi A., Ahmad M., Mabood F., Hassan Z., Shah A., Green I.R., Schulz B. (2015). Microsphaerol and seimatorone: Two new compounds isolated from the endophytic fungi, *Microsphaeropsis* sp. and *Seimatosporium* sp.. Chem. Biodivers..

[84] Blunt J.W., Copp B.R., Keyzers R.A., Munro M.H.G., Prinsep M.R. (2014). Marine natural products. Nat. Prod. Rep..

[85] Imhoff J.F. (2016). Natural products from marine fungi—Still an underrepresented resource. Mar. Drugs.

[86] Williams R.B., Henrikson J.C., Hoover A.R., Lee A.E., Cichewicz R.H. (2008). Epigenetic remodeling of the fungal secondary metabolome. Org. Biomol. Chem..

[87] Chung Y., Wei C., Chuang D., El-Shazly M., Hsieh C.T., Asai T., Oshima Y., Hsieh T.J., Hwang T.L., Wu Y. (2013). An epigenetic modifier enhances the production of anti-diabetic and anti-inflammatory sesquiterpenoids from *Aspergillus sydowii*. Bioorg. Med. Chem..

[88] Beau J., Mahid N., Burda W.N., Harrington L., Shaw L.N., Mutka T., Kyle D.E., Barisic B., Olphen A., Baker B.J. (2012). Epigenetic tailoring for the production of anti- infective cytosporones from the marine fungus *Leucostoma persoonii*. Mar. Drugs.

[89] Bhatnagar I., Kim S.K. (2010). Immense essence of excellence: Marine microbial bioactive compounds. Mar. Drugs.

[90] Duarte K., Rocha-Santos T.A.P., Freitas A.C., Duarte A.C. (2012). Analytical techniques for discovery of bioactive compounds from marine fungi. Trends Anal. Chem..

